# Usability of an At-Home Anterior Nares SARS-CoV-2 RT-PCR Sample Collection Kit: Human Factors Feasibility Study

**DOI:** 10.2196/29234

**Published:** 2021-12-14

**Authors:** Laura E Strong, Irene Middendorf, Michelle Turner, David K Edwards V, Varun Sama, Joshua Mou, K Colleen Adams

**Affiliations:** 1 Exact Sciences Corporation Madison, WI United States

**Keywords:** COVID-19 testing, at-home collection kit, SARS-CoV-2, feasibility studies, self-collection, usability study, COVID-19

## Abstract

**Background:**

Readily available testing for SARS-CoV-2 is necessary to mitigate COVID-19 disease outbreaks. At-home collection kits, in which samples are self-collected without requiring a laboratory or clinic visit and sent to an external laboratory for testing, can provide convenient testing to those with barriers to access. They can prevent unnecessary exposure between patient and clinical staff, increase access for patients with disabilities or remote workers, and decrease burdens on health care resources, such as provider time and personal protective equipment. Exact Sciences developed an at-home collection kit for samples to be tested to detect SARS-CoV-2 that includes an Instructions for Use (IFU) document, which guides people without prior experience on collecting a nasal swab sample. Demonstrating successful sample collection and usability is critical to ensure that these samples meet the same high-quality sample collection standards as samples collected in clinics.

**Objective:**

The aim of this study was to determine the usability of a SARS-CoV-2 at-home nasal swab sample collection kit.

**Methods:**

A human factors usability study was conducted with 30 subjects without prior medical, laboratory, or health care training and without COVID-19 sample self-collection experience. Subjects were observed while they followed the IFU for the at-home sample collection portion of the SARS-CoV-2 test in a setting that simulated a home environment. IFU usability was further evaluated by requiring the subjects to complete a survey, answer comprehension questions, provide written feedback, and respond to questions from the observer about problems during use.

**Results:**

All 30 subjects successfully completed the sample collection process, and all 30 samples were determined by reverse transcription–polymerase chain reaction (RT-PCR) testing to meet quality standards for SARS-CoV-2 testing. The subjects’ written feedback and comments revealed several recommendations to improve the IFU.

**Conclusions:**

The study demonstrated the overall usability of an at-home SARS-CoV-2 collection kit. Various feedback mechanisms provided opportunities to improve the wording and graphics for some critical tasks, including placing the label correctly on the tube. A modified IFU was prepared based on study outcomes.

## Introduction

The global pandemic caused by SARS-CoV-2 has resulted in 223 million confirmed cases of COVID-19, including 4.6 million deaths, as of September 2021, according to the World Health Organization [1]. In the United States, there have been more than 40.3 million reported COVID-19 cases and more than 649,000 deaths as of September 2021 [1]. The transmission of COVID-19 has been shown to be contained with a combination of isolation practices, including wearing masks, physical distancing, and lockdown measures [2], and widespread immunization with effective COVID-19 vaccines [3]. In the United States, more than 175 million people have been fully vaccinated as of September 2021 using one of three COVID-19 vaccines [4-6] currently authorized for emergency use by the US Food and Drug Administration (FDA)—in August 2021, the FDA approved Comirnaty, known previously as the Pfizer-BioNTech COVID-19 vaccine, for individuals 16 years of age or older [7].

Despite this monumental progress, significant challenges remain to manage the ongoing pandemic. Many eligible Americans have not been vaccinated [1], and the emergence of increasingly virulent strains, including the Delta variant [8], have resulted in increased hospitalizations and deaths throughout the United States [9,10]. Widespread testing can help public health officials to better monitor the progression of the pandemic, identify emerging variants, and identify individuals with COVID-19, particularly those with asymptomatic disease.

One approach to broadening access to SARS-CoV-2 testing has been the development of at-home sample collection kits that could be used safely and effectively by people without medical or laboratory experience. Samples can be collected without needing to travel to a medical center, and the samples can be shipped and later processed at a laboratory or health care facility or tested at home [11]. At-home sample collection offers multiple advantages to combat the COVID-19 pandemic: it can prevent unnecessary exposure between patients and clinical staff during collection; improve access for elderly patients, patients with disabilities, or remote workers; reduce the need for personal protective equipment; and shift the logistics of collection from overburdened clinical sites to commercial delivery services.

Surveys on the perception of at-home COVID-19 sample collection and tests have demonstrated a broad willingness to complete such collection and confidence in the sample preparation and quality [12]. Multiple studies have been conducted recently to compare SARS-CoV-2 test results from self-collected samples to those collected by health care workers [13-18]. Recently, a large-scale population-based study on the applicability of COVID-19 self-testing demonstrated that most participants collected the sample correctly the first time, and that test results showed comparable performance to those collected by health care professionals [19].

To investigate the usability of an at-home collection process, usability studies should be conducted to ensure that these samples meet the same quality standards as clinician-collected samples. Here, we describe the results of a human factors usability study, conducted early in the pandemic, for the at-home sample collection kit, herein referred to as the “SARS-CoV-2 at-home collection kit,” for use with the SARS-CoV-2 (N gene detection) Test, both of which were developed by Exact Sciences. Exact Sciences is a molecular diagnostics company that manufactures an at-home screening test for colorectal cancer and developed the SARS-CoV-2 at-home collection kit and test in response to the global pandemic. Additional details related to the SARS-CoV-2 test are available in the FDA’s Emergency Use Authorization (EUA) documentation in Multimedia Appendix 1. 

The goal of this study was to determine the usability of the SARS-CoV-2 at-home collection kit, and our primary endpoint was the percentage of samples collected from study participants that returned a valid SARS-CoV-2 result.

This study was conducted in May 2020, during the early months of the pandemic when very little information about COVID-19 pathogenesis was available, and the protocol was designed based on standards for human factors usability study design [20]. By publishing our methodology and outcomes, we hope to provide a blueprint for future studies to ensure that the usability of other at-home collection kits can be quickly evaluated during public health crises or similar situations where urgency is required.

## Methods

### SARS-CoV-2 Test

The SARS-CoV-2 (N gene detection) Test was developed by Exact Sciences and received EUA from the FDA on May 22, 2020, via EUA200367 (Multimedia Appendix 1). A summary of the SARS-CoV-2 test characteristics is provided in Table 1. This is a reverse transcription–polymerase chain reaction (RT-PCR)–based test that evaluates upper respiratory samples, including those collected with an anterior nares (ie, nasal) swab, to detect regions within the nucleocapsid (N) gene of the novel coronavirus (nCoV), specifically the nCoV_N1 and nCoV_N2 regions. Human ribonuclease P (RNase P), a gene expressed ubiquitously in human cells regardless of COVID-19 infection, serves as a control to demonstrate that usable samples were collected and provided to the lab, and that all testing processes were successfully completed. In validation studies, the test demonstrated no cross-reactivity with a panel of known respiratory pathogens. Its preclinical test performance in a collection of test samples showed positive percent agreement of 95% (38 out of 40 samples) and negative percent agreement of 100% (38 out of 38 samples) with another FDA-authorized SARS-CoV-2 RT-PCR-based test.

The SARS-CoV-2 at-home collection kit contains the following: a sterile, individually wrapped nasal swab with a polyester tip with plastic handle; a 2-mL transport tube containing 0.9% saline; an Instructions for Use (IFU) document; a biohazard bag; an absorbent pad; a specimen identification label; and a UN3373-labeled shipping container.

**Table 1:** Performance of the SARS-CoV-2 detection test from Exact Sciences.

Test^a^ characteristic		Details
Test name		SARS-CoV-2 (N gene detection) Test
Type of test		Real-time RT-PCR^b^
**Gene regions detected**		
	SARS-CoV-2	nCoV^c^_N1 and nCoV_N2 regions of the nucleocapsid (N) gene
	Control	Ribonuclease P human gene locus
Limit of detection		2.6 genome copies/µL sample
Cross-reactivity		13 other respiratory pathogens not detected^d^
**Preclinical test performance^e^ (n=78 samples)**		
	Positive percent agreement (38 out of 40 samples), % (95% CI)	95.0 (83.5-98.6)
	Negative percent agreement (38 out of 38 samples), % (95% CI)	100 (90.8-100)

^a^Test details were obtained from the US Food and Drug Administration (FDA) Emergency Use Authorization (EUA) summary (Multimedia Appendix 1).

^b^RT-PCR: reverse transcription–polymerase chain reaction.

^c^nCoV: novel coronavirus.

^d^The detection assay was conducted using NATtrol Respiratory Pathogen Panel-1 (NATRPP-1) from Zeptometrix.

^e^In comparison with another COVID-19 RT-PCR test with FDA EUA.

### Study Objectives and Subjects

The main objective was to determine the usability of the SARS-CoV-2 at-home collection kit for the collection and mailing of a nasal swab sample to the testing laboratory. The primary endpoint was the percentage of samples from the study participants that returned a valid SARS-CoV-2 test result, either positive or negative, both of which require a detectable level of RNase P. The target percentage was 80%, given that the subjects were minimally trained and were inexperienced in sample self-collection. There were five secondary objectives: (1) evaluate the perceived usability of the IFU, (2) evaluate the comprehension of the IFU by the subject, (3) identify problems that occur while following the IFU, (4) evaluate the root causes of problems that occur while following the IFU, and (5) develop strategies to mitigate problems occurring while following the IFU. A total of 30 patients from a workforce population that met established inclusion and exclusion criteria were enrolled in this study. Steps were taken to recruit subjects of varying ages and educational statuses, which included a manual review of participants by the study team.

The study was conducted in accordance with state and federal regulatory requirements, as well as the general principles set forth in the International Ethical Guidelines for Biomedical Research Involving Human Subjects [21] and the Declaration of Helsinki [22]. The study was approved by the Western Institutional Review Board (WIRB)–Copernicus Group Institutional Review Board (No. 20201763) and all subjects provided written informed consent prior to study participation.

The inclusion criterion was the ability to provide informed consent, and the exclusion criteria were prior medical or laboratory training, prior experience with COVID-19 sample self-collection, and prior SARS-CoV-2 testing. For each enrolled subject, usability of the IFU was determined based on successful completion of the self-collection of a nasal swab sample, which included a valid SARS-CoV-2 test result. All subjects completed the study.

### Study Design

**Figure 1:**
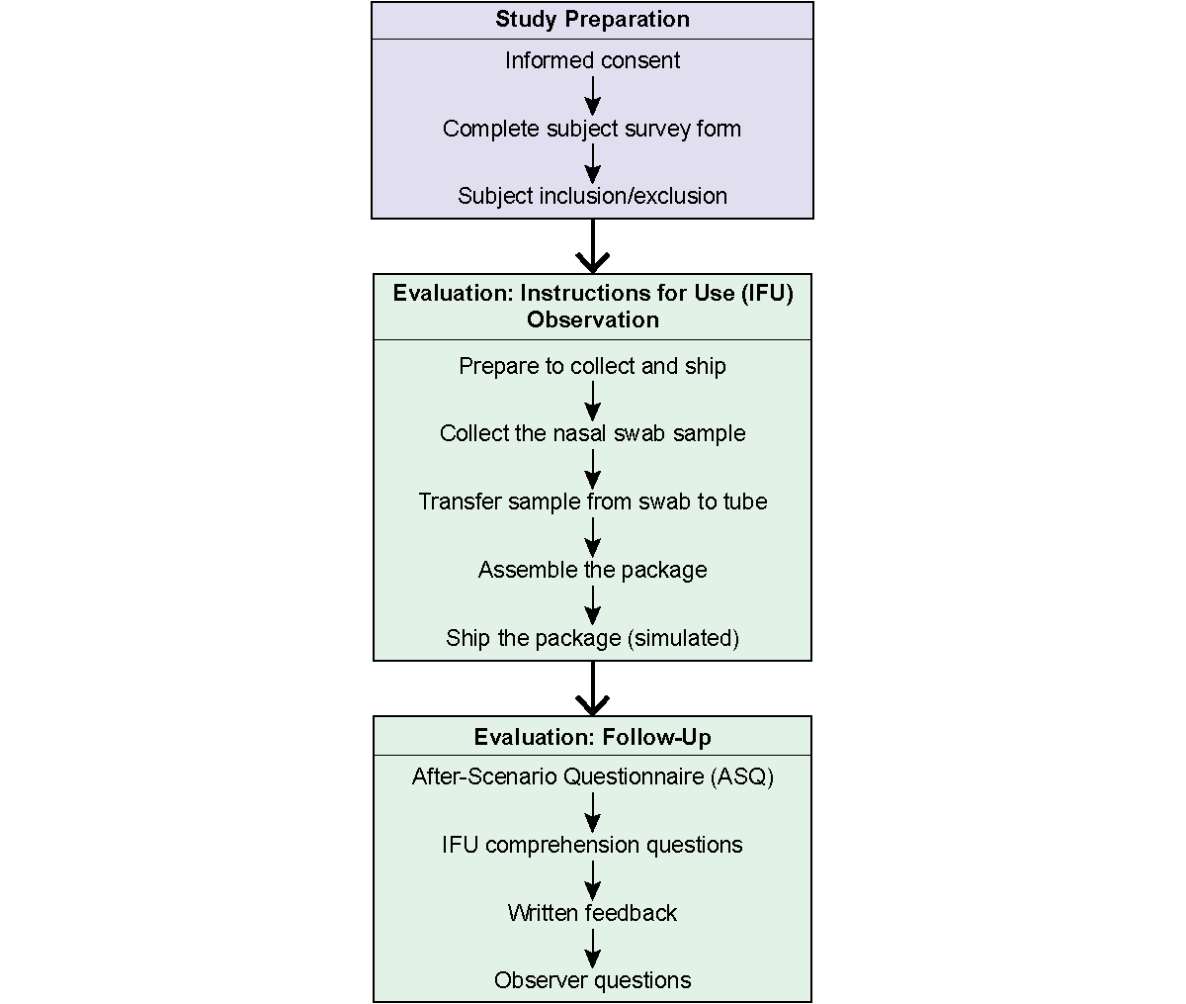
Overview of human factors usability study procedures.

A use-related Failure Mode Effects Analysis approach was used to determine potential hazards and their associated risks during use of the SARS-CoV-2 at-home collection kit. Subjects completed a survey form with demographic information, including race and ethnicity [23], highest education level obtained, and prior experience with medical or laboratory training and COVID-19 sample self-collection. Subjects who provided informed consent were provided an overview of the clinical study procedures, including guidance that they would be observed during the sample collection and answer questions related to their experience during the sample collection. The study consisted of two parts: simulated use, in which sample collection was simulated by a subject while monitored by an observer; and postsimulation evaluation, in which the subject completed survey questions and provided feedback on the collection process. The overall study design and methodology is summarized in Figure 1.

The sample collection took place in a simulated home environment in a conference room with a table that served as a large surface area, similar to a countertop found in a kitchen or bathroom. The room included common household items, such as hand sanitizer, pens, pencils, paper towels, and a wastebasket. Since a sink was not available in the room, a large bowl labelled “SINK” was provided next to the hand sanitizer to simulate a sink for handwashing with soap and water.

Before beginning the sample collection, the observer oriented the subject to the simulated environment, making them aware of items available to them, without indicating that these would be required for the sample collection to reduce bias to the subject. To begin the sample collection, the observer instructed the subject to retrieve an available kit and to begin the collection; subjects were then observed while following the IFU for the SARS-CoV-2 at-home collection kit. The identification and classification of tasks were made by the study researchers (see Figure 1 for complete list of tasks), and the study participants were blinded to the task categories.

Following completion of all steps in the IFU, the subject was then instructed to place the sample package in a designated area within the conference room. The subject then provided feedback on the usability of the IFU by completing the After-Scenario Questionnaire (ASQ) [24-26], answered comprehension questions, provided written feedback on the experience, and addressed questions from the observer about observed problems during use.

The samples collected by the subjects were tested for SARS-CoV-2 by Exact Sciences Laboratories. Subjects remained blinded to the test results.

## Results

The study was conducted using the SARS-CoV-2 at-home collection kit from Exact Sciences; test characteristics are summarized in Table 1 (Multimedia Appendix 1). Briefly, the laboratory test used RT-PCR to detect SARS-CoV-2 from a sample collected using an anterior nares (ie, nasal) swab, using detection of human RNase P as a control.

For the human factors usability study, 30 subjects were enrolled to simulate at-home sample collection and provide feedback during the follow-up evaluation (Figure 1). The characteristics of the study subjects (N=30) are described in Table 2. The mean age of the subjects was 38.0 (SD 9.7) years, and no subjects were older than 65 years. Most subjects were White (n=26, 87%) and non-Hispanic or non-Latino (n=25, 83%), and 77% (n=23) of subjects had more than a high school education. After completing the usability study, all 30 subjects’ self-collected samples resulted in a valid SARS-CoV-2 test result, and all were negative for SARS-CoV-2. Sample validity was determined by successful detection of human RNase P.

Subjects completed the simulated sample collection according to an IFU document that described the 26 tasks required to prepare, collect, and ship a nasal swab sample (see Multimedia Appendix 2 for complete list of tasks). These tasks were divided into “critical tasks,” in which use errors or failure to complete would have a negative clinical impact, such as invalid or delayed test results, and “essential tasks,” which were important for test completion but did not pose an immediate risk to the sample. Out of the 26 tasks, 15 were categorized as “critical” and were the primary focus for evaluating and improving the IFU based on study outcomes and subject feedback.

**Figure 2:**
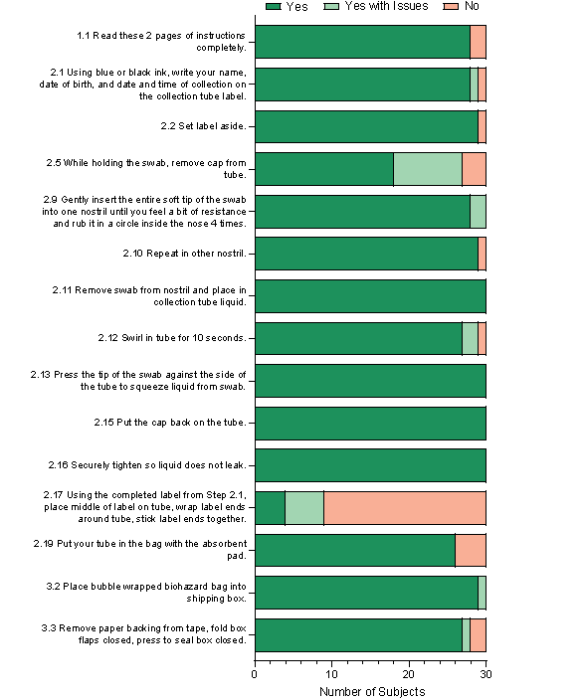
Observation of success in completing critical tasks using the SARS-CoV-2 at-home collection kit.

Overall, 14 out of 15 critical tasks from the IFU were successfully completed by more than 80% of the subjects during the simulated sample collection (Figure 2). The task that the subjects encountered the greatest difficulty with was placing the label on the tube, which was not completed properly by 70% (21/30) of the subjects.

**Table 2:** Subject characteristics for human factors usability study.

Characteristic			Value (N=30)
Age (years), mean (SD)			38.0 (9.7)
**Age category (years), n (%)**			
	<18	0 (0)	
	18-30	9 (30)	
	31-45	12 (40)	
	46-65	9 (30)	
	>65	0 (0)	
**Sex, n (%)**			
	Female	18 (60)	
	Male	12 (40)	
**Ethnicity, n (%)**			
	Hispanic or Latino	4 (13)	
	Non-Hispanic or non-Latino	25 (83)	
	Unknown	1 (3)	
**Race^a^, n (%)**			
	American Indian or Alaska Native	0 (0)	
	Asian	2 (7)	
	Black or African American	2 (7)	
	Native Hawaiian or other Pacific Islander	0 (0)	
	White	26 (87)	
	Unknown	0 (0)	
**Education level, n (%)**			
	No high school	0 (0)	
	Some high school	0 (0)	
	High school degree only	7 (23)	
	College degree	18 (60)	
	Advanced degree	5 (17)	

^a^Subjects had the option to report one or more categories for race; each participant selected an option for both ethnicity and race.

To evaluate the opinions of the subjects after the simulated sample collection, subjects completed the ASQ (Table 3) [26]. Responses were indicated on a scale of 1 to 7, with lower scores corresponding to higher satisfaction; scores of less than 3 indicated that subjects felt satisfied using the IFU for sample collection. For all questions among the 30 subjects, the mean overall ASQ score was 2.1 (SD 1.6), indicating overall satisfaction.

The subjects’ written feedback and comments to the observer revealed several areas of potential improvement to the IFU. Most comments focused on references to the front and back of the IFU, handwashing, handling the absorbent pad, how far into the nostrils the nasal swab should be inserted (eg, use of the word “resistance”), and issues related to the tube label (eg, writing on the label and attaching it to the tube).

**Figure 3:**
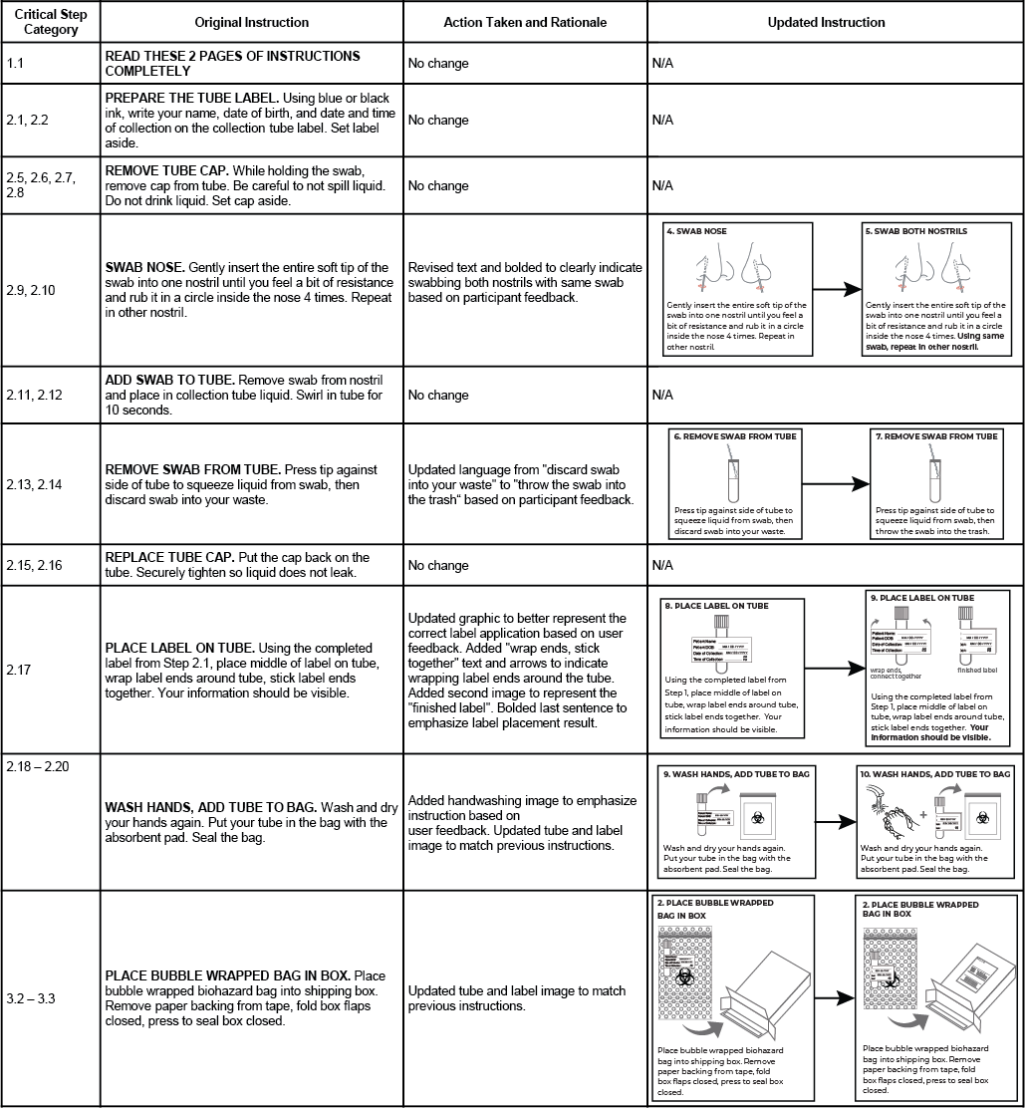
Revised Instructions for Use based on human factors usability study results. N/A: not applicable.

Results from the multiple sources of feedback collected during the feasibility study (Table 3) were combined to determine how to improve the usability of the IFU (see Figure 3 for complete list of changes and corresponding rationale). This feedback helped improve the language and graphics describing how to place the label on the tube, which is the critical task with the lowest successful completion rate. Furthermore, subjects suggested minor wording changes to improve comprehension (eg, replacing “discard swab into your waste” with “throw swab into the trash”). The updated IFU is shown in Multimedia Appendix 3.

**Table 3:** Methods of evaluating Instructions for Use (IFU) sample collection and shipping tasks for SARS-CoV-2 at-home collection kit by the observer. After-Scenario Questionnaire (ASQ) questions were from Lewis [26].

Feedback	Categories or questions	Measurement
Observer evaluation of IFU	Reading the instructions (1 step) Preparing for collection (2 steps) Preparing the tube label (2 steps) Opening nasal swab (2 steps) Removing the tube cap (4 steps) Swabbing nose (2 steps) Adding swab to tube (2 steps) Removing swab from tube (2 steps) Replacing the tube cap (2 steps) Placing label on tube (1 step) Washing hands and adding tube to bag (3 steps) Placing bag in bubble wrap (1 step) Placing bubble-wrapped bag in box (2 steps)	The observer selected one of four options: Subject completed task with no issues Subject completed task with issues or unexpected effort Subject did not complete task or required assistance Not applicable (subject discontinued participation).
ASQ questions	ASQ1. Overall, I am satisfied with the ease of completing the tasks in this scenario. ASQ2. Overall, I am satisfied with the amount of time it took to complete the tasks in this scenario. ASQ3. Overall, I am satisfied with the support information (online help, messages, and documentation) when completing the tasks.	The subject recorded their response on a numerical 7-point scale, ranging from 1 (“strongly agree”) to 7 (“strongly disagree”), and “N/A” (not applicable) outside the scale.
IFU comprehension	After collecting a nasal swab sample, when should a person ship it to the lab? How should a person store the package with the nasal swab sample inside before shipping it back to the lab? What could happen to your nasal swab sample if you do not follow the steps in the instructions for use?	The observer recorded the response as “correct” or “incorrect” (with the option to record free-form text and ask follow-up questions).
Written feedback	What information in the IFU is confusing? Is there anything we could do to make it easier to collect a nasal swab sample using these materials?	The observer recorded the response as free-form text (with the option to ask follow-up questions).
Observer questions	Did the subject experience or report adverse events? Were any protocol deviations noted? Did the subject complete the study?	The observer recorded the response as “yes” or “no.”

## Discussion

Overall, this simulated at-home self-collection usability study was successful in that all 30 subjects collected samples that resulted in valid test results (100% success rate, exceeding the targeted 80%). Moreover, the ASQ scores were low, indicating acceptable agreement and satisfaction, and the written feedback and comments from subjects were combined with simulation data to improve the IFU for future patients undergoing COVID-19 testing using at-home specimen collection.

To evaluate sample quality, the presence of human RNase P, a gene expressed ubiquitously in human cells regardless of COVID-19 infection, provided a universal measurement of quality control. If the sample contained a detectable level of RNase P, then it was determined that the sample had sufficient RNA to be tested for the presence of SARS-CoV-2. Notably, this standard can be used for any sample obtained using the at-home SARS-CoV-2 collection kit, regardless of the method of collection or the positive or negative outcome of the test. The preclinical test characteristics of the SARS-CoV-2 test, summarized in Table 1, demonstrated high positive and negative percent agreement among samples of sufficient quality.

The study population was well distributed with respect to age and gender. The proportion with a college degree or higher (77%) was slightly higher than the local population in Madison, Wisconsin (58%), although race and ethnicity populations were similar (ie, the White, non-Hispanic or non-Latino population in Madison is 74%) [27]. Importantly, the qualifications for study participants impacted the inclusion criteria, which required the exclusion of anyone with any scientific or laboratory experience.

In general, the ability to provide at-home sample collection to detect respiratory viruses could significantly improve the effectiveness of public health strategies in preventing the spread of disease during a pandemic. For the COVID-19 pandemic, at-home sample collection could (1) improve the ability to identify individuals with detectable SARS-CoV-2 RNA without the need to expose health care workers during testing or the public during travel to and from the testing site, (2) provide an alternative and likely more accessible testing workflow for patients, and (3) enable the epidemiological study of the natural history of disease without undue risk to the population.

This study had several limitations. Some limitations were the simulated nature of the home environment, the lack of access to shipment methods for subjects, and the lack of subjects over 65 years, which was a result of the workforce population recruited for the study. Other limitations, including the relatively small sample size, were based on limited access of materials and a prioritization to make this collection kit available as soon as possible due to the ongoing public health crisis. Changes driven by logistics or product considerations and typographical errors are included in the updated IFU but are beyond the scope of this publication. The strengths of this study were that all subjects were able to successfully follow the IFU to collect usable samples, the consistency of the completion of medium-risk tasks, and the constructive feedback on low-risk tasks that led to IFU improvements.

In conclusion, this study demonstrated the overall usability of the SARS-CoV-2 at-home collection kit, and feedback from the study was used to generate improved instructions for use. Overall, it provides additional information that at-home collection of specimens for use with COVID-19 tests can be conducted effectively by subjects without prior sample self-collection experience.

## Appendix

Multimedia Appendix 1 US Food and Drug Administration Emergency Use Authorization documentation for the Exact Sciences SARS-CoV-2 test.

Multimedia Appendix 2 List of steps in Instructions for Use for the SARS-CoV-2 at-home collection kit. Critical steps are indicated in bold, which were not uniquely highlighted or bolded to subjects during the study.

Multimedia Appendix 3 Final Instructions for Use document.

## Abbreviations

ASQ: After-Scenario Questionnaire
EUA: Emergency Use Authorization
FDA: Food and Drug Administration
IFU: Instructions for Use
N: nucleocapsid
nCoV: novel coronavirus
RNase P: ribonuclease P
RT-PCR: reverse transcription–polymerase chain reaction
WIRB: Western Institutional Review Board
