# Supplementary material for: Usability of an At-Home Anterior Nares SARS-CoV-2 RT-PCR Sample Collection Kit: Human Factors Feasibility Study
Source: JMIR Hum Factors. 2021 Dec 14;8(4):e29234. doi: 10.2196/29234 (PMC8673714; doi:10.2196/29234)
Supplement: Multimedia Appendix 1 [file humanfactors_v8i4e29234_app1.pdf]

EMERGENCY USE AUTHORIZATION (EUA) SUMMARY

SARS-CoV-2 (N gene detection) Test

(Exact Sciences Laboratories)

For *In vitro* Diagnostic Use
Rx Only
For use under Emergency Use Authorization (EUA) only

The SARS-CoV-2 RT-PCR assay will be performed at the Exact Sciences Laboratories, located at 650 Forward Drive, Madison, WI 53711 and 145 E. Badger Road Ste. 100, Madison, WI 53713, which are certified under the Clinical Laboratory Improvement Amendments of 1988(CLIA), 42 U.S.C. §263a and meet the requirements to perform high complexity tests as per Laboratory Standard Operating Procedures that were reviewed by the FDA under this EUA.

UPDATED SUMMARY ON FDA WEBSITE

INTENDED USE

The *SARS-CoV-2 (N gene detection) Test* is a high-throughput real-time RT-PCR assay intended for the qualitative detection of nucleic acid from SARS-CoV-2 in upper respiratory specimens (such as nasal, mid-turbinate, nasopharyngeal, and oropharyngeal swab specimens), from individuals suspected of COVID-19 by a healthcare provider. Testing is limited to Exact Sciences Laboratories, located at 650 Forward Drive, Madison, WI 53711 and 145 E. Badger Road Ste. 100, Madison, WI 53713, which are certified under the Clinical Laboratory Improvement Amendments of 1988 (CLIA), 42 U.S.C. § 263a, and meet the requirements to perform high complexity tests.

This test is also for use with nasal swab specimens that are self-collected at home or in a healthcare setting by individuals using an authorized home-collection kit specified in this EUA's authorized labeling when determined to be appropriate by a healthcare provider.

Results are for the detection of SARS-CoV-2 RNA. The SARS-CoV-2 RNA is generally detectable in respiratory specimens during the acute phase of infection. Positive results are indicative of the presence of SARS-CoV-2 RNA; clinical correlation with patient history and other diagnostic information is necessary to determine patient infection status. Positive results do not rule out bacterial infection or co-infection with other viruses. The agent detected may not be the definite cause of disease.

Laboratories within the United States and its territories are required to report all positive results to the appropriate public health authorities.

Negative results do not preclude SARS-CoV-2 infection and should not be used as the sole basis for patient management decisions. Negative results must be combined with clinical observations, patient history, and epidemiological information.

The *SARS-CoV-2 (N gene detection) Test* is intended for use by clinical laboratory personnel specifically instructed and trained in the techniques of real-time RT-PCR assays. The assay is intended for use under the Food and Drug Administration's Emergency Use Authorization.

DEVICE DESCRIPTION AND TEST PRINCIPLE

The SARS-CoV-2 (N gene detection) Test is a high-throughput real-time reverse transcription polymerase chain reaction (rRT-PCR) test with a testing capacity of 120,000 tests per week. The test is run biplexed into a single reaction and amplification setup using a specific primer and probe set to detect one region (nCOV_N1) in the SARS-CoV-2 nucleocapsid (N) gene and one primer and probe set to detect human RNase P (RP) in a clinical sample. RNA isolated from upper respiratory specimens (i.e., nasopharyngeal, oropharyngeal or nasal swab) is reverse transcribed to cDNA and subsequently amplified using the ABI 7500 Fast Dx (Applied Biosystems) instrument with Sequence Detection Software (SDS) version 1.4.1. During the amplification process, the probe anneals to a specific target sequence located between the forward and reverse primers. During the extension phase of the PCR cycle, the 5' nuclease activity of Taq polymerase degrades the bound probe, causing the reporter dyes (FAM and Q610) to separate from the quencher dye (BHQ), generating a fluorescent signal. Fluorescence intensity is monitored at each PCR cycle by the ABI 7500 Fast Dx.

INSTRUMENTS USED WITH TEST

RNA extraction based on conventional well-known bead-based technology is conducted using the non-commercial, Exact Sciences Corporation extraction procedure on the Hamilton STARlet liquid handler. RT-PCR is performed on the ABI 7500 Fast Dx (Applied Biosystems) real-time PCR instrument.

COLLECTION KITS USED WITH TEST

The *SARS-CoV-2 (N gene detection) Test* is to be used with the following home collection kit:

- Everlywell COVID-19 Test Home Collection Kit – to self-collect nasal swab specimens when determined to be appropriate by a healthcare provider. Testing of self-collected nasal swabs is performed according to the Accessioning SOP and Instructions for Use of the Everlywell COVID-19 Test Home Collection Kit. Everlywell has granted Exact Sciences Laboratories a right of reference to the data supporting the use of this authorized home collection kit.
- Exact Sciences COVID-19 Nasal Swab Home-Collection Kit consisting of a separately packed dry sterile swab (synthetic-tipped nasal swab with plastic or aluminum shaft) a 2mL collection tube with 1.2 mL 0.9% saline, collection tube label, biohazard bag with absorbent pad, bubble wrap and shipping box.

UPDATED SUMMARY ON FDA WEBSITE

UPDATED SUMMARY ON FDA WEBSITE

MEDICAL OVERSIGHT OF THE SELF-COLLECTION AND RESULT REPORTING PROCESS

Medical Oversight of the self-collected sample testing process is provided by the healthcare providers. Patients will be evaluated by the healthcare provider and determined to be appropriate for at-home collection based on current CDC screening guidelines. Once at-home collection eligibility is determined, an order is placed by the physician (this equals prescription).

When the Everlywell Collection Kit is used, the order is placed with Everlywell. The Everlywell Collection Kits are shipped to the patients by Everlywell but have return labels that will directly ship the collected samples to Exact Sciences Laboratories. In this case the HCP will put the order in directly with Everlywell. Results will be reported to Everlywell and they will report it to the provider per their SOP.

When the Exact Sciences Collection Kit is used, the order is placed by the healthcare provider with Exact Sciences Laboratories. The Exact Sciences Collection Kits are shipped to the patients with return labels addressed to Exact Sciences Laboratories.

After specimen collection by the patient the samples are shipped to Exact Sciences. All at-home collection specimens arriving at Exact Sciences Laboratories (ESL) will be inspected for the following rejection criteria:

- Specimens received >56 hours after collection
- Specimen label is not present on specimen tube or does not include two patient identifiers
- Specimen is grossly contaminated
- Transport tube is broken or leaking
- No transport media is within the collection tube resulting in no sample for testing

The Test results are communicated back by Exact Sciences Laboratories to the healthcare provider. The healthcare provider is responsible for reporting the result and next steps to the patient.

EQUIPMENT, REAGENTS AND MATERIALS

The following equipment/reagents/materials are required to run this test:

1. Exact Sciences Corporation extraction reagents
2. Hamilton STARlet liquid handler for nucleic acid extraction
3. ABI 7500 Fast Dx (Applied Biosystems) for cDNA synthesis and PCR amplification of the target sequences.
4. GoTaq 1-Step RT-qPCR system (Promega; #A6121)
5. 2019-nCoV N1 and RP primers and probes (Exact Sciences Laboratories; #10006626)
6. Hs_RPP30 Positive Control (RP) (IDT; #10006626)
7. 2019-nCoV_N_Positive Control (N1 & N2) (IDT; #10006625)

CONTROLS TO BE USED WITH THE COVID-19 RT-PCR

- Extraction No Target (ENT) serves both as an extraction control to monitor for any Reagent contamination and sample carryover that could occur during the extraction process. The ENT consists of Saline (ESL specimen collection media) and is run once for every batch of extracted specimens.

- A no template control (NTC) is used to monitor the possibility of sample contamination in the assay run and is used once on every PCR assay plate. The control is DNA suspension buffer (TE buffer).

- A Positive Control (POC) (nCoVPC) is used to verify that the assay run is performing as intended. The nCoVPC contains targets for N1 and RP and consists of Hs_RPP30 Positive Control (IDT) and 2019-nCoV_N_Positive Control (IDT) in DNA suspension buffer (TE buffer). The positive control is used once on every PCR plate.

- Internal Control: Each sample that contains nucleic acid must demonstrate the presence of the internal control (IC) amplicon. The IC is created from PCR amplification of a locus within the RNase P human gene and monitors adequate amounts and quality of RNA in the sample and correct sample processing.

INTERPRETATION OF RESULTS

All test controls should be examined prior to interpretation of patient results. If the controls are not valid, the patient results cannot be interpreted.

a. Control Result Interpretation

Table 1: Expected Performance of Controls

Control	2019 nCoV_N1	RP	Expected Ct Values
ENT	-	-	RP: No Ct, or 10*> Ct > 37;<br>N1: 8* >Ct > 40
NTC	-	-	RP: No Ct, or 10*> Ct > 37;<br>N1: 8* >Ct > 40
nCoVPC (POS)	+	+	RP: 10* ≤ Ct ≤ 37<br>N1: 8* ≤ Ct ≤ 40

If any of the above controls do not exhibit the expected performance as described, the assay may have been improperly set up and/or executed improperly, or reagent or equipment malfunction could have occurred. Invalidate the run and re-test.

UPDATED SUMMARY ON FDA WEBSITE

b. Examination and Interpretation of Patient Specimen Results:

Assessment of clinical specimen test results should be performed after the positive and negative controls have been examined and determined to be valid and acceptable. If the controls are not valid, the patient results cannot be interpreted.

Table 2: Interpretation of Patient Results

N1	RP	Result Interpretation	Patient Report Verbiage
+	+/-	All targets are valid. SARS-CoV-2 (COVID-19) RNA detected.	SARS-CoV-2 (COVID-19) RNA detected
-	+	All targets are valid. SARS-CoV-2 (COVID-19) RNA **NOT** detected.	SARS-CoV-2 (COVID-19) RNA **NOT** detected. Negative results do not preclude SARS-CoV-2 (COVID-19) infection and should not be used as the sole basis for treatment or other patient management decisions.
-	-	Results are invalid.<br>Repeat testing<br>If the result is still invalid, a new specimen should be obtained.	**Invalid** – This specimen exhibited inhibition in the PCR assay or the specimen contained an inadequate amount of clinical material. Repeat testing is suggested if clinically indicated.

PERFORMANCE EVALUATION

1) *Analytical Sensitivity:*

The LoD of the SARS-CoV-2 (N gene detection) Test was determined using the AccuPlex SARS-CoV-2 Reference Material Kit (SeraCare). AccuPlex recombinant materials are constructed with a replication-deficient mammalian virus producing a safe, non-infectious material. These mammalian virus-based reference materials resemble the complexity of virus targets found in true patient samples, including the viral particle protein coat and lipid bilayer.

a. Tentative LoD

The tentative LoD for the N1 target within the SARS-CoV-2 genome was determined using 2-fold serial dilutions with 20 to 29 replicates per dilution using known titers (genome copies (cp)/µL) of AccuPlex SARS-CoV-2 Positive Reference Material (SeraCare) in negative nasal swab matrix collected in saline. Spiked samples were tested with the SARS-CoV-2 (N gene detection) Test following the laboratory SOP.

Results are summarized in Table 3. The LoD was determined as the lowest concentration where ≥ 95% of the replicates were positive. Accordingly, the N1 gene has an LoD of 1.2 copies/µL of specimen (**Table 3**).

UPDATED SUMMARY ON FDA WEBSITE

Table 3: SARS-CoV-2 Tentative LoD

Target Level	Valid results	N 1 Positive		SARS-CoV-2 (N 1) Detection Rate	RP Positive		SARS-CoV-2 (RP) Detection Rate
		n	Mean Ct		n	Mean Ct	
4.8 cp/µL	3	3	34.5	100%	3	26.9	100%
2.4 cp/µL	3	3	35.6	100%	3	26.9	100%
**1.2 cp/µL**	**3**	**3**	**36.7**	**100%**	**3**	**27.0**	**100%**
0.6 cp/µL	3	3	38.4	67%	3	26.8	100%
0.3 cp/µL	3	3	38.1	67%	3	26.8	100%
0.15 cp/µL	3	3	38.4	33%	3	26.9	100%

b. Confirmatory LoD

The LoD was confirmed by testing additional 20 replicates at 1x LoD. Dilutions were generated using AccuPlex SARS-CoV-2 Reference Material (SeraCare) spiked into a pool of negative nasal swab specimens in UTM to mimic clinical specimens. Spiked samples were tested with the SARS-CoV-2 (N gene detection) Test following the laboratory SOP. Results are summarized in Table 4.

The LoD of the SARS-CoV-2 (N gene detection) Test was determined to be 1.2 copies/µl.

Table 4: Confirmatory LoD

	N 1 gene	RP (Internal Control)
**RNA concentration – 1.2 cp/µL of sample**		
**Positives**	20/20	20/20
**Mean Ct**	36.3	26.6
**Std Dev (Ct)**	1	0.1

2) ***Analytical Inclusivity/Specificity:***

a. Inclusivity

The SARS-CoV-2 (N gene detection) Test utilizes primer and probe sets identical to the N1 SARS-CoV-2 target genes used in the CDC 2019-Novel Coronavirus (2019-CoV) Real-Time RT-PCR Diagnostic Panel. Accordingly, an *in silico* inclusivity analysis was not repeated.

UPDATED SUMMARY ON FDA WEBSITE

b. Cross-Reactivity

i. *In Silico* Analysis

The SARS-CoV-2 (N gene detection) Test utilizes primer and probe sets identical to the N1 SARS-CoV-2 target genes used in the CDC 2019-Novel Coronavirus (2019-CoV) Real-Time RT-PCR Diagnostic Panel. Accordingly, an *in silico* cross-reactivity analysis was not repeated.

ii. Wet Testing

Wet testing was performed by Exact Sciences Laboratories to further evaluate the specificity of the SARS-CoV-2 (N gene detection) Test. Studies were performed using the NATtrol Respiratory Pathogen Panel-1 (NATRPP-1) from Zeptometrix, pools 1-5, which is formulated with purified, intact virus particles and bacterial cells that have been chemically modified to render them non-infectious. Samples were tested according to the laboratory SOP. No signal was detected for the N1 gene for any of the tested replicates (n=1) of any of the organisms included in the analysis (Table 5).

Table 5. Organisms Tested for Cross-Reactivity

Pool	Respiratory Virus/Bacteria	N1 Gene
1	Influenza A H1N1 (A/NY/02/09)	Not detected
1	Parainfluenza Type 4A	Not detected
1	Parainfluenza Type 4B	Not detected
1	Rhinovirus (1A)	Not detected
1	Adenovirus Type 3	Not detected
2	Influenza A H1 (A/New Caledonia/20/99)	Not detected
2	Respiratory Syncytial Virus A	Not detected
2	Parainfluenza Type 1	Not detected
2	Coronavirus NL63	Not detected
2	Mycoplasma pneumoniae (M129)	Not detected
3	Influenza A H3 (A/Brisbane/10/07)	Not detected
3	Respiratory Syncytial Virus B (CH93(18)-18)	Not detected
3	Coronavirus OC43	Not detected
3	Coronavirus HKU	Not detected
4	Influenza B (B/Florida/02/06)	Not detected
4	Parainfluenza Type 3	Not detected
4	Human Metapneumovirus (Peru6-2003)	Not detected
4	*Legionella pneumophila*	Not detected
5	Parainfluenza Type 2	Not detected
5	Coronavirus 229E	Not detected
5	Human Bocavirus	Not detected
5	*Chlamydophila pneumoniae* (CWL-029)	Not detected

UPDATED SUMMARY ON FDA WEBSITE

c. Interfering Substances

The SARS-CoV-2 (N gene detection) Test (biplex format) was evaluated for susceptibility to potential exogenous and endogenous interferents associated with upper respiratory infections and specimens respectively. A negative patient specimen pool in Saline was spiked with AccuPlex SARS-CoV-2 Positive Reference Material at 1-2X LoD (+ SARS-CoV-2 Target) or combined with a negative patient specimen pool (- SARS-CoV-2 Target). All samples were processed in the presence or absence of interfering substances at the concentrations noted in Table 6. None of the substances evaluated had an adverse effect on the ability of the assay to detect the SARS-CoV-2 target.

Table 6: Interference Study with Mucin and Blood

SARS-CoV-2 Target	Substance	Concentration	Number of Replicates	Mean Ct		Interference
				N1	RP	
+	Control	N/A	3	34.4	27.7	N/A
	Afrin	15% v/v	3	36.7	29.5	No
	Allergy Nasal Spray	5% v/v	3	35.2	27.4	No
	Blood	5% v/v	3	34.4	25.9	No
	Bovine Mucin[1]	2.5 mg/mL	3	34.3	30.6	No
	Cepacol throat lozenges (menthol + Benzocaine)	5 mg/mL	3	34.8	27.5	No
-	Control	N/A	3	NA	26.8	N/A
	Afrin	15% v/v	3	NA	30.1	No
	Allergy Nasal Spray	5% v/v	3	NA	26.9	No
	Blood	5% v/v	3	NA	25.7	No
	Bovine Mucin[1]	2.5 mg/mL	3	NA	28.1	No
	Cepacol throat lozenges (menthol + Benzocaine)	5 mg/mL	3	NA	26.9	No

[1]Bovine submaxillary glands Type I-S (Sigma-Aldrich; Cat. No. M3895)

3) Clinical Evaluation:

A total of 120 de-identified nasal swab specimens collected in saline were used for clinical evaluation. All samples were tested with the SARS-CoV-2 (N gene detection) Test (biplex format) and concordance was determined based on results obtained with the authorized singleplex format of the same test (N1, N2 and RP). The data indicate 100% concordance between the singleplex and biplex test results for a clinical sample. Concordance was based on a Ct value < 40 for specimens considered positive and a Ct value of ≥ 40 for specimens considered negative for the presence of SARS-CoV-2 N1 gene. Results are summarized in Table 7.

UPDATED SUMMARY ON FDA WEBSITE

Note: Because an inconclusive result indicates that one of the test targets is concordant with the comparator while the other target is discordant with the comparator, inconclusive results are excluded from performance calculation. The rate of inconclusive results is 0.83% (1/120).

Table 7: Evaluation with Clinical Specimens

		**SARS-CoV-2 (N gene detection) Test (Singleplex format)**		
		Positive	Inconclusive	Negative
**SARS-CoV-2 (N gene detection) Test (Biplex format)**	Positive	52	1*	0
	Negative	0	0	67

* The inconclusive specimen was positive for N2 with a Ct value of 39.9 indicative of a low positive sample right at the cutoff (i.e., Ct 40), the original assay was negative for N1 with the original EUA test. The A002 bi-plex version containing only the N1 target was positive with a Ct value of 38.4.

For the clinical sample testing PPA and NPA after exclusion of the two invalid specimens and the Wilson Score Method based 95% Confidence Intervals were as follows:
Positive Percent Agreement (PPA): 52/52 = 100% (95% CI: 93.1% - 100%)
Negative Percent Agreement (NPA): 67/67 = 100% (95% CI: 94.6% - 100%)

4) ***Studies to support Home-Collection with the Exact Sciences Collection Kit***

a. Usability Study

The usability study evaluated subject's ability to successfully complete self-collection of a nasal swab sample, including a valid SARS-CoV-2 test result as defined by a positive RNase P signal in all samples. The study consisted of three part: (a) the subjects performed the entire self-collection, packing and mock-shipping process according to the intended collection instructions in a simulated at-home environment without assistance, (b) Observation during the self-collection with recording of failures, and (c) feedback from the study participants. Participants were of varying ages and educational background and had no former experience in self-collecting specimens. Any prior medical experience and prior experience with self-collecting samples excluded the individual from the study.

Test Results (RNase P)
All subjects (30 out of 30) had valid test results, which were negative for SARS-CoV-2 and positive for RNase P. All samples had strong Human RNase P signals indicating all participants were successful in collecting human biological material. No sample had to be rejected due to missing labels, lost volume or improper packaging.

Observations
Most steps in the IFU were followed easily by more than 75% of subjects. The least "failures" were observed in the actual swab collection and the shipping/packaging. The tasks with the highest failure rates (by observation) were related to how to hold the swab

UPDATED SUMMARY ON FDA WEBSITE

and remove the cap at the same time and to affixation of the patient label on the tube without overlapping the ends. These instructions have been revised to address these failures.

After-Collection Questionnaire
All 30 subjects completed the After-Survey Questionnaire (ASQ) and sored their satisfaction with the collection instructions. A score of 1 is given to the highest satisfaction, a score of 7 is given to the highest dissatisfaction. Scores of 3 or lower indicate that the subject agreed with being satisfied using the IFU for sample collection. Individuals reported a mean overall score of 2.1 with a standard deviation of 1.6. The subjects also provided written feedback and comment that have been incorporated into an updated IFU.

b. Shipping Stability

FDA has reviewed analytical validation data from a swab stability study conducted by Quantigen Biosciences, with support from The Gates Foundation and UnitedHealth Group, that the sponsor references to support sample stability of synthetic tipped nasal swab shipped in saline (0.9%) for testing with the authorized Exact Sciences SARS-CoV-2 test. Quantigen Biosciences has granted a right of reference to any sponsor wishing to pursue an EUA to leverage their COVID-19 swab stability data as part of that sponsor's EUA request.

c. Robustness of Swab Elution

Because the collection device does not use swab with breakable shafts, this study is aimed to assess the limited elution time of the swab in the saline transport solution and the impact of related operator steps (insertion of the swab, swirling in the transport media and pressing the swab against the wall when removing it for disposal).
Pooled nasal matrix and AccuPlex SARS-CoV-2 Positive Reference Material (SeraCare) were loaded onto fresh swabs that are part of the collection convenience kit. The positive reference material was added at a concentration of 2x and 5x LoD in pooled negative nasal matrix. After preparing the swabs they were eluted in the saline containing transport tube as described in the Table below. Each condition was tested with 3 replicates each at 2x and 5x LoD.

Table 8. Detection of RP and N1 targets with varying elution methods for nasal swab samples spiked at 2X or 5X LoD with SARS-CoV-2 positive reference material.

Elution Method	Detection at 2x LoD (X/3)		Detection at 5x LoD (X/3)	
	RP	N1	RP	N1
A. Per IFU instructions: Swirling for 10 seconds plus squeezing against the tube wall	3/3	3/3	3/3	3/3
B. Vigorous shaking of the swab	3/3	3/3	3/3	3/3
C. Swirling only 3 seconds with squeezing against the wall	3/3	3/3	3/3	3/3

UPDATED SUMMARY ON FDA WEBSITE

Elution Method	Detection at 2x LoD (X/3)		Detection at 5x LoD (X/3)	
	RP	N1	RP	N1
D. Swirling only 3 seconds without squeezing against the wall	3/3	3/3	3/3	3/3
E. No swirling (just a dip in and take out of the swab with no agitation in the saline) with squeezing against the wall	3/3	1/3	3/3	3/3
F. No swirling (just a dip in and take out of the swab with no agitation in the saline) without squeezing against the wall	3/3	2/3	3/3	3/3

The study demonstrated that samples with a very low virus concentration below 5x LoD may yield false negative results if the swab is only briefly dipped into the solution. However, the collection instructions mitigate this user error by a prominently placed warning on the first page that no result may be obtained when the correct procedure is not followed, and by bolding the most critical steps.

LIMITATIONS:

- For in vitro diagnostic use
- For prescription use only
- For use under FDA emergency use authorization (EUA) only
- Members of the infectious disease laboratory will be trained to perform this assay and competency will be assessed and documented per CAP regulations.
- The SARS-CoV-2 (N gene detection) Test has not been FDA cleared or approved;
- The test has been authorized by FDA under an Emergency Use Authorization (EUA) for use by Exact Sciences Laboratories, which are certified under CLIA and meet the requirements to perform high complexity tests;
- The SARS-CoV-2 (N gene detection) Test has been authorized only for the detection of nucleic acid from SARS-CoV-2, not for any other viruses or pathogens.
- The SARS-CoV-2 (N gene detection) Test is only authorized for the duration of the declaration that circumstances exist justifying the authorization of emergency use of *in vitro* diagnostic tests for detection and/or diagnosis of COVID-19 under Section 564(b)(1) of the Federal Food, Drug and Cosmetic Act, 21 U.S.C. § 360bbb-3(b)(1), unless the authorization is terminated or revoked sooner.
- The performance of this SARS-CoV-2 assay was established using Nasal swab specimens. Nasopharyngeal, oropharyngeal and mid-turbinate swabs and BAL are also considered acceptable specimen types for use with the SARS-CoV-2 assay, but performance has not been established.
- A false negative result may occur if a specimen is improperly collected, transported or handled. False negative results may also occur if amplification inhibitors are present in the specimen or if inadequate numbers of organisms are present in the specimen.
- This test cannot rule out diseases caused by other bacterial or viral pathogens.

UPDATED SUMMARY ON FDA WEBSITE
