# Supplementary material for: Usability of an At-Home Anterior Nares SARS-CoV-2 RT-PCR Sample Collection Kit: Human Factors Feasibility Study
Source: JMIR Hum Factors. 2021 Dec 14;8(4):e29234. doi: 10.2196/29234 (PMC8673714; doi:10.2196/29234)
Supplement: Multimedia Appendix 2 [file humanfactors_v8i4e29234_app2.pdf]

*1 Before you collect*

**1.1 Read these 2 pages of instructions completely**

1.2 Wash your hands with soap and water. Dry your hands with a clean towel.

1.3 Put all collection materials on a clean dry surface. Refer to page 2 for kit contents.

*2 Collect your nasal swab sample*

**2.1 Using blue or black ink, write your name, date of birth, and date and time of collection on the collection tube label.**

**2.2 Set label aside**

2.3 Remove the nasal swab from the wrapper by pulling the two ends of the wrapper apart.

2.4 Be careful to only touch the swab handle, not the swab tip

**2.5 While holding the swab, remove cap from tube.**

2.6 Be careful to not spill liquid.

2.7 Do not drink liquid.

2.8 Set cap aside.

**2.9 Gently insert the entire soft tip of the swab into one nostril until you feel a bit of resistance and rub it in a circle inside the nose 4 times.**

**2.10 Repeat in other nostril.**

**2.11 Remove swab from nostril and place in collection tube liquid.**

**2.12 Swirl in tube for 10 seconds.**

**2.13 Press the tip of the swab against the side of the tube to squeeze liquid from swab.**

2.14 Discard swab into your waste.

**2.15 Put the cap back on the tube.**

**2.16 Securely tighten so liquid does not leak.**

**2.17 Using the completed label from Step 2.1, place middle of label on tube, wrap label ends around tube, stick label ends together.**

2.18 Wash and dry your hands again.

**2.19 Put your tube in the bag with the absorbent pad.**

2.20 Seal the bag.

*3 Ship your kit back to the lab*

3.1 Fold the bubble wrap around sealed biohazard bag.

**3.2 Place bubble wrapped biohazard bag into shipping box.**

**3.3 Remove paper backing from tape, fold box flaps closed, press to seal box closed.**
