# Supplementary material for: Usability of an At-Home Anterior Nares SARS-CoV-2 RT-PCR Sample Collection Kit: Human Factors Feasibility Study
Source: JMIR Hum Factors. 2021 Dec 14;8(4):e29234. doi: 10.2196/29234 (PMC8673714; doi:10.2196/29234)
Supplement: Multimedia Appendix 3 [file humanfactors_v8i4e29234_app3.pdf]

DocuSign Envelope ID: 0D21943E-10AD-47FE-AD20-FE6301EC948C

Rx only. For Emergency Use Authorization only.
For in vitro diagnostic use only. For use by people 18 years of age and older.

Nasal Swab Self-Collection Instructions (COVID-19 Virus Detection Test)

BEFORE YOU COLLECT

- **READ THESE 2 PAGES OF INSTRUCTIONS COMPLETELY**
- **PLAN YOUR COLLECTION TIME**
  Ship your sample on the same day as your collection. **Do not collect and ship your sample on Saturday or Sunday.**
  Call 1-800-823-7459 to schedule a pick-up or drop off at a UPS drop box location before the last pickup of the day. The package is postage paid and pre-addressed.

COLLECT YOUR NASAL SWAB SAMPLE

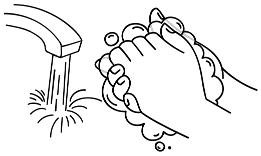

1. WASH YOUR HANDS WITH SOAP AND WATER

Dry your hands with a clean towel. Put all collection materials on a clean dry surface. Refer to back side of document for kit contents.

2. PREPARE THE TUBE LABEL

Patient Name: ____________
Patient DOB: MM / DD / YYYY
Date of Collection: MM / DD / YYYY
Time of Collection: ____________ AM PM

Using blue or black ink, write your name, date of birth, and date and time of collection on the Collection Tube Label. Set label aside.

3. OPEN NASAL SWAB

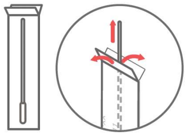

Remove the nasal swab from the wrapper by pulling the two ends of the wrapper apart. Be careful to only touch the swab handle, not the swab tip.

4. REMOVE TUBE CAP

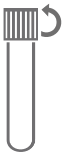

While holding the swab, remove cap from tube. Be careful to not spill liquid. Do not drink liquid. Set cap aside.

5. SWAB BOTH NOSTRILS

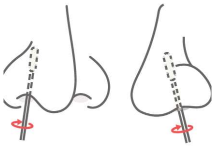

Gently insert the entire soft tip of the swab into one nostril until you feel a bit of resistance and rub it in a circle inside the nose 4 times. **Using same swab, repeat in other nostril.**

6. ADD SWAB TO TUBE

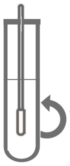

Remove swab from nostril and place in collection tube liquid. Swirl in tube for 10 seconds.

7. REMOVE SWAB FROM TUBE

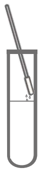

Press tip against side of tube to squeeze liquid from swab, then throw the swab into the trash.

8. REPLACE TUBE CAP

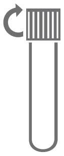

Put the cap back on the tube. Securely tighten so liquid does not leak.

9. PLACE LABEL ON TUBE

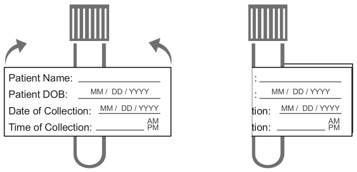

Using the completed label from Step 1, place middle of label on tube, wrap label ends around tube, stick label ends together. **Your information should be visible.**

10. WASH HANDS, ADD TUBE TO BAG

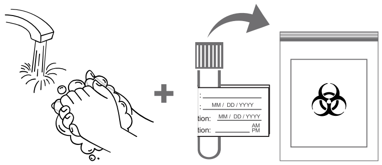

Wash and dry your hands again. Put your tube in the bag with the absorbent pad. Seal the bag.

Turn over to back side of document for shipping instructions.

Turn over to back side of document for shipping instructions.

EXACT SCIENCES V3, 06-2020

Images 3 and 5 courtesy of ©2020 Audere

SHIP YOUR KIT BACK TO THE LAB

PLEASE READ:

- Ship your sample on the same day as your collection. Do not collect and ship your sample on Saturday or Sunday.
- Store sample in sealed box away from direct heat and sunlight until shipment.

1. PLACE BAG IN BUBBLE WRAP

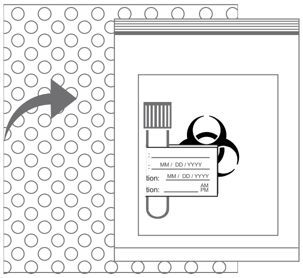

Fold the bubble wrap around sealed biohazard bag.

2. PLACE BUBBLE WRAPPED BAG IN BOX

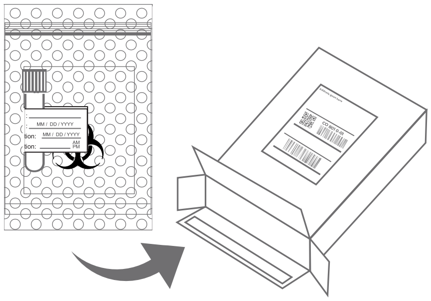

Place bubble wrapped biohazard bag into shipping box. Remove paper backing from tape, fold box flaps closed, press to seal box closed.

3. PREPARE YOUR PACKAGE FOR SHIPMENT

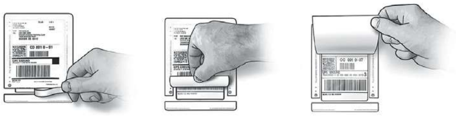

The UPS shipping label has a green tear strip that must be removed.

- Lift the left corner of the tear strip and pull it off.
- Lift the bottom edge of the label and pull up.
- Tear the top label off the kit along the perforation.
- Your return label shows.
- You are ready to ship your package.
- Continue with the next step.

4. SHIP PACKAGE BY UPS

Option 1: Call UPS special returns line:
1-800-823-7459 to schedule a pick-up

- When UPS asks for the company you are shipping to, say **"At Home with Exact"**
- Provide the tracking number shown on your prepaid return shipping label, which starts with **1Z**.

Option 2: Drop your kit off at a UPS drop box before the last pickup of the day.
Visit www.ups.com/dropoff to locate the nearest drop box and pickup hours.

DO NOT schedule your pickup via UPS.com.

IMPORTANT!

To ensure your sample is processed by the lab, you must:

- Complete and apply the sample label to the tube with your name and date of birth.
- Ensure that the tube is completely closed after the nasal swab collection.
- Ship your package on the same day as your collection. Do not collect and ship your sample on Saturday or Sunday.

Your sample may not be processed and you may not receive a test result if you do not follow these steps.

COLLECTION KIT CONTENTS

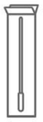

Nasal Swab

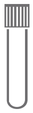

Collection Tube (with 0.9% saline)

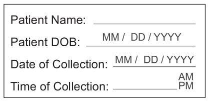

Collection Tube Label

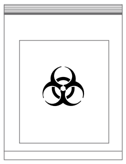

Biohazard Bag (with Absorbent Pad)

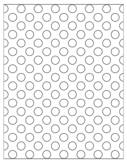

Bubble Wrap

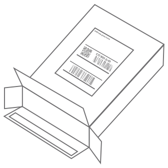

Shipping Box

EXACT SCIENCES V3, 06-2020

650 Forward Drive | Madison, WI 53711
844.570.9730 | www.exactlabs.com

©2020 Exact Sciences Corporation. All rights reserved.
